# Supplementary material for: Uncovering the balance of forces driving microtubule aster migration in C. elegans zygotes
Source: Nat Commun. 2018 Mar 5;9:938. doi: 10.1038/s41467-018-03118-x (PMC5838244; doi:10.1038/s41467-018-03118-x)
Supplement: Supplementary file 1 — Supplementary Information [file 41467_2018_3118_MOESM1_ESM.pdf]

Supplementary Figures

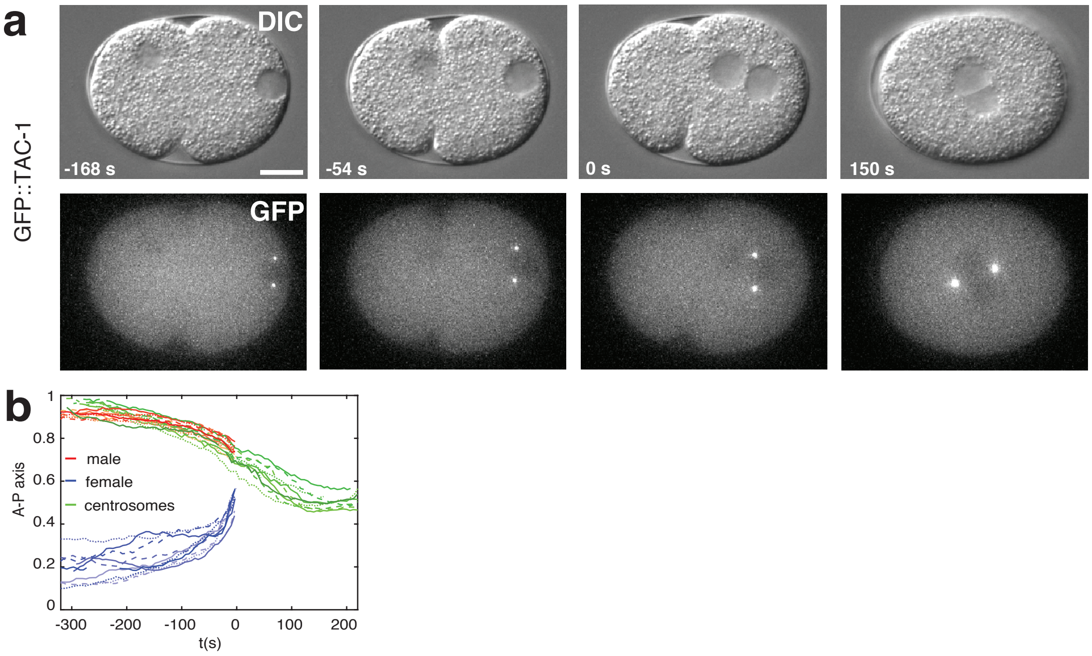

Supplementary Figure 1

Imaging pronuclear migration and centration in one-cell *C. elegans* embryo.

a DIC (top panels) and GFP fluorescence microscopy (bottom panels – z-maximum projections) of embryo expressing GFP::TAC-1. Time is indicated in seconds and time 0 s is pronuclear meeting. Scale bar: 10 µm.

b Pronuclear and centrosome midpoint positions along the A-P axis as a function of time for 10 representative GFP::TAC-1 embryos. Here and in other Supplementary Figures, position on the A-P axis is represented in normalized coordinates (0: anterior; 1: posterior).

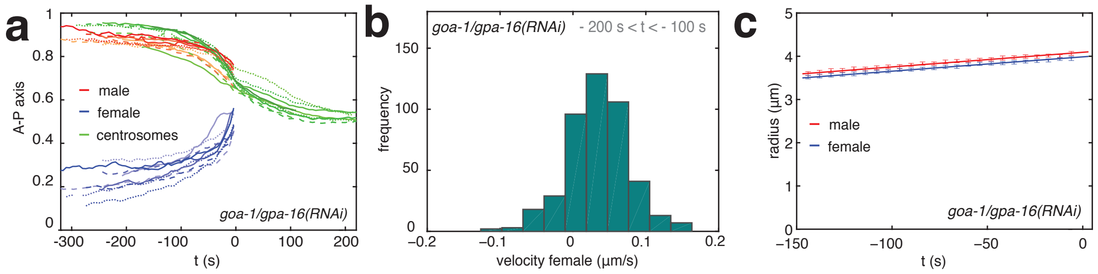

Supplementary Figure 2

Pronuclear and centrosome midpoint positions, as well as pronuclear velocities during the slow migration phase and pronuclei radii in *goa-1/gpa-16*(*RNAi*) embryos.

a Pronuclear and centrosome midpoint positions along the A-P axis as a function of time for 10 representative *goa-1/gpa-16*(*RNAi*) embryos.

b Distribution of female pronucleus velocities during the slow phase of migration in *goa-1/gpa-16*(*RNAi*) embryos (-200 s < t < -100 s; n = 31; Kolmogorov-Smirnov test (two-sided) P = 0.56; mean: 0.034 ± 0.002 μm s^{-1}; SD: 0.042 ± 0.001 μm s^{-1}).

c Pronuclear radii as a function of time in *goa-1/gpa-16(RNAi)* embryos with SEM (n = 31). Red and blue lines: linear fit (male – slope: 0.0034 ± 0.0001 μm s^{-1}, offset: 4.09 ± 0.02 μm; female - slope: 0.0033 ± 0.0001 μm s^{-1}, offset: 3.99 ± 0.01 μm; errors are S.D.).

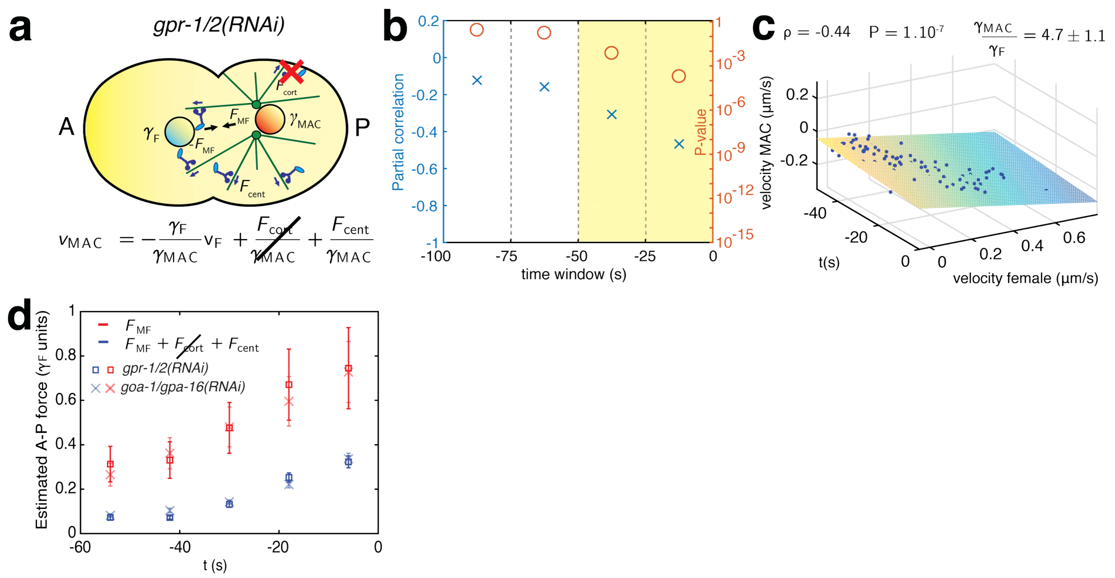

Supplementary Figure 3

Pronuclear migration and centration upon depletion of cortical forces in *gpr-1/2(RNAi)* embryos.

a Schematics of pronuclear migration and centration in *gpr-1/2*(*RNAi*) embryos. Represented are the female and male pronuclei (blue and red disks, respectively), centrosomes (green dots), microtubules (green lines), dynein motors (blue) and dynein anchors (light blue ellipses); arrows represent exerted forces. Anterior (A) and posterior (P) sides are indicated. F_{MF}: force exerted between the male-asters complex and the female pronucleus; F_{cort}: force exerted by cortical dynein; F_{cent}: centering force; γ_{F}: drag coefficient of the female pronucleus; γ_{MAC}: drag coefficient of the male-asters complex. The red cross indicates depleted cortical dynein motors.

b Partial Pearson's correlation, controlling for time-variation, between pronuclear velocities along the A-P axis over successive time windows in *gpr-1/2(RNAi)* embryos (n = 20). Each time-point corresponds to a time window of 25 s and the correlated time window (P < 0.05) is highlighted in yellow (partial correlation: blue crosses; P-value (Student's t-test, two-sided): orange circles). Here and in panel c, velocities are calculated between successive frames 6 s apart.

c Velocity of male pronucleus as a function of time and of velocity of the female pronucleus during the correlated phase of pronuclear migration in *gpr-1/2(RNAi)* embryos (n = 20, -50 < t < 0 s time-window). Plane: linear fit $v_{MAC} = -\frac{\gamma_F}{\gamma_{MAC}} v_F + v_0 + mt$ ($v_0 = (-0.11 \pm 0.02)\ \mu m\ s^{-1}$; $m = (-\ 0.001 \pm 0.0005) \mu m\ s^{-2}$; errors are S.D.). The Pearson partial correlation coefficient ρ between the velocities of the male-asters complex and female pronucleus, controlling for time variation, its P-value (Student's t-test, two-sided) and the fitted ratio between the drag coefficients of the male-asters complex and female pronucleus are indicated.

d A-P forces acting on the male-asters complex and female pronuclei shortly before pronuclear meeting with SEM. Blue: force between pronuclei, estimated from the force acting on the female pronucleus in *gpr-12(RNAi)* embryos (blue squares, n = 20). The force is compatible with that determined in *goa-1/gpa-16*(*RNAi*) embryos (blue crosses, n = 31; χ^2 = 9; P = 0.11) embryos. Red: sum of the forces acting between pronuclei and the centering force, estimated as that acting on the male-asters complex in *gpr-12(RNAi)* (red squares, n= 20). The force is compatible with that measured in *goa-1/gpa-16*(*RNAi*) embryos (red crosses, n = 31; χ^2 = 0.5; P = 0.99).

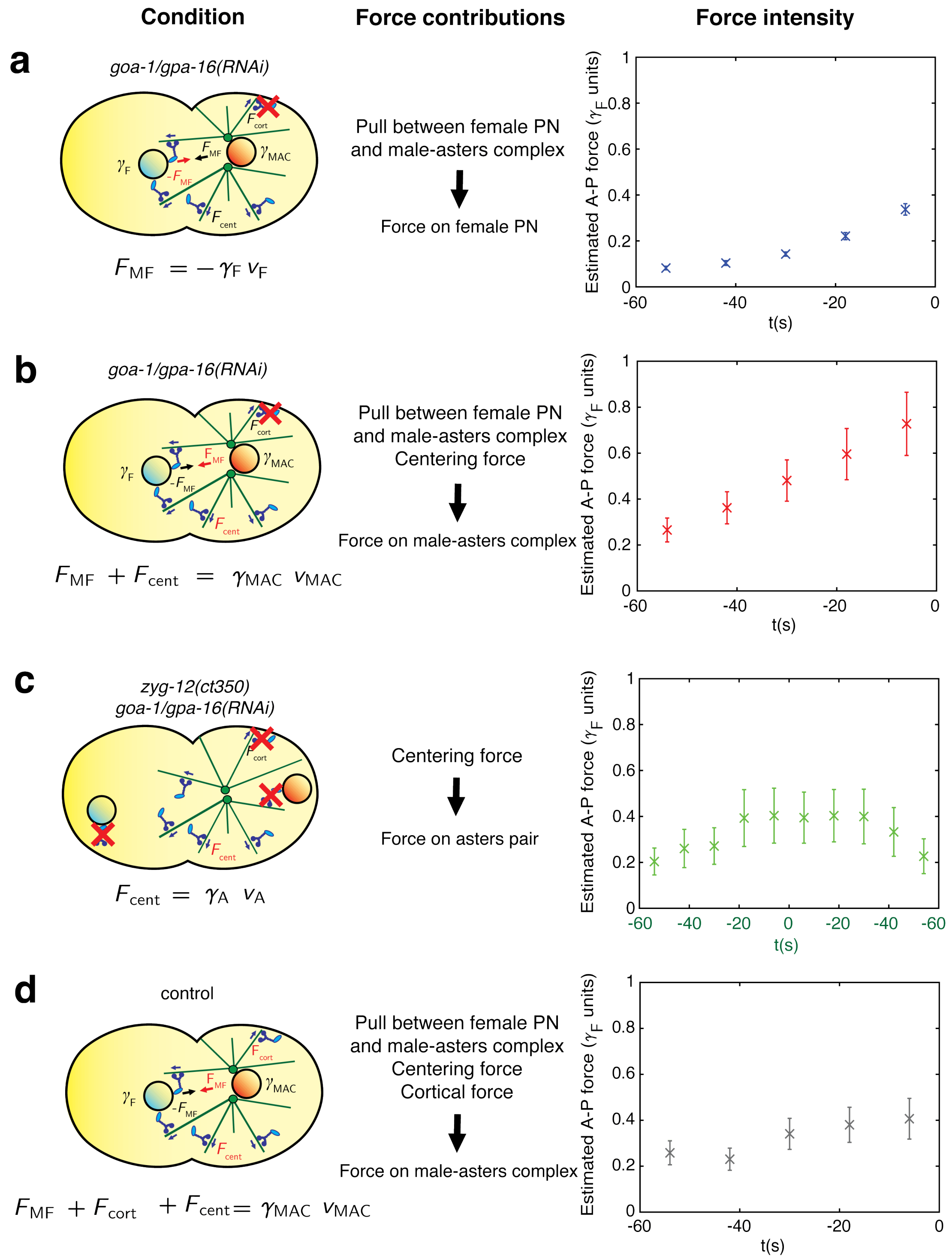

Supplementary Figure 4

Overview of forces acting in pronuclear migration and centration in mutant/RNAi and control embryos.

a Schematics (left) and intensity (right, A-P component, with SEM, n = 31) of the time evolution of the total force acting on the female pronucleus in *goa-1/gpa-16(RNAi)* embryos, corresponding to the force exerted between the male-asters complex and the female pronucleus. Here, as well as in b and in d, time 0 s is pronuclear meeting. The data presented in this figure recapitulates that presented in Fig. 1, 3 and 4.

b Schematics (left) and intensity (right, A-P component, with SEM, n = 31) of the time evolution of the total force acting on the male-asters complex in *goa-1/goa-16(RNAi)* embryos, corresponding to the sum of the force exerted between the male-asters complex and the female pronucleus, plus the centering force.

c Schematics (left) and intensity (right, A-P component, with SEM, n = 8) of the time evolution of the centering force in *zyg-12(ct350) goa-1/goa-16(RNAi)* embryos, which corresponds to the total force acting on the asters pair. Time 0 s is taken as the fitted half-centration time (indicated by the green lettering on the x-axis; Methods).

d Schematics (left) and intensity (A-P component, right) of the total force acting on the male-asters complex in control embryos (right, A-P component, with SEM, n = 33), corresponding to the sum of the force exerted between the male-asters complex and the female pronucleus, plus the centering force, as well as forces mediated by cortical dynein.

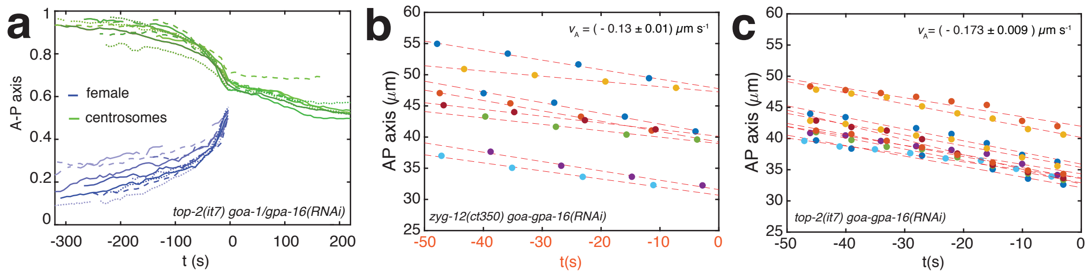

Supplementary Figure 5

Pronuclear and centrosome midpoint positions in *top-2(it7) goa-1/gpa-16*(*RNAi*) embryos, as well as comparison of centrosome midpoint velocities in *zyg-12(ct350) goa-1/gpa-16(RNAi)* and *top-2(it7) goa-1/gpa-16(RNAi)* embryos.

a Centrosome midpoint positions along the A-P axis as a function of time for 10 representative *top-2(it7) goa-1/gpa-16*(*RNAi*) embryos.

b, c Comparison of centrosome midpoint velocities in *zyg-12(ct350) goa-1/gpa-16(RNAi)* (b, n = 8 embryos) and *top-2(it7) goa-1/gpa-16(RNAi)* embryos (c, n = 10 embryos). In *top-2(it7) goa-1/gpa-16(RNAi)* embryos, time 0 s is defined at pronuclear meeting. In *zyg-12(ct350) goa-1/gpa-16(RNAi)* embryos, since pronuclear meeting is not occurring, time 0 s is defined at the time at which centrosomes reach on average the same position that they reach in *top-2(it7) goa-1/gpa-16(RNAi)* embryos at pronuclear meeting (67% of AP-axis length; indicated by the orange lettering on the x-axis). Dots of different colors represent centrosome midpoint positions in different embryos. The slope of the A-P trajectory of the asters pair of each embryo in the -50 s < t < 0 s time-window is fitted with a linear function (dashed red lines). The average asters pair velocity v_A is calculated by averaging the slopes calculated for each individual embryo and for each condition. The average asters pair velocity is higher in *top-2(it7) goa-1/gpa-16(RNAi)* than in *zyg-12(ct350) goa-1/gpa-16(RNAi)* embryos (z-test; P = 0.007 (two-sided)).

Supplementary Table 1

	Model	Interaction MAC- female PN	Time-dependency	Variable drag	AIC
1	$V_{\mathrm{MAC}} = V_0$	-	-	-	136
2	$V_{\mathrm{MAC}} = V_0 + mt$	-	+ (linear)	-	62
3	$V_{\mathrm{MAC}} = V_0 + mt + nt^2$	-	+ (up to quadratic)	-	64
4	$V_{\mathrm{MAC}} = V_0 - \frac{\gamma_{\mathrm{F}}}{\gamma_{\mathrm{MAC}}} V_{\mathrm{F}}$	+ (linear)	-	-	19
**5**	$\boldsymbol{V_{\mathrm{MAC}} = V_0 - \frac{\gamma_{\mathrm{F}}}{\gamma_{\mathrm{MAC}}} V_{\mathrm{F}} + mt}$ **(Eq.3)**	**+ (linear)**	**+ (linear)**	**-**	**16**
6	$V_{\mathrm{MAC}} = V_0 - \frac{\gamma_{\mathrm{F}}}{\gamma_{\mathrm{MAC}}} V_{\mathrm{F}} + mt + nt^2$	+ (linear)	+ (up to quadratic)	-	20
7	$V_{\mathrm{MAC}} = V_0 - \frac{\gamma_{\mathrm{F}}}{\gamma_{\mathrm{MAC}}} V_{\mathrm{F}} + b\, {V_{\mathrm{F}}}^2$	+ (up to quadratic)	-	-	20
8	$V_{\mathrm{MAC}} = V_0 - \frac{\gamma_{\mathrm{F}}}{\gamma_{\mathrm{MAC}}} V_{\mathrm{F}} + b\, {V_{\mathrm{F}}}^2 + mt$	+ (up to quadratic)	+ (linear)	-	17
9	$V_{\mathrm{MAC}} = V_0 - \frac{\gamma_{\mathrm{F}}}{\gamma_{\mathrm{MAC(0)}}} V_{\mathrm{F}} + r' t\, V_{\mathrm{F}} + mt$	+ (linear)	+ (linear)	+ (*)	17

Model selection analysis by the Akaike Information Criterion

Distinct models of the relationship between the velocity of the male-asters complex, that of the female pronucleus and time compared by the Akaike Information Criterion (AIC). The table reports whether the interaction between the male-asters complex (MAC) and female pronucleus (female PN), the time-dependency of the centering force, as well as a variable drag coefficient of the male-asters complex, are included in each model. The model that fits the dataset with highest quality, i.e. the one with the lowest AIC, is Model 5 (Eq. 3). (*): in Model 9, the drag coefficient of the male-asters complex varies linearly with time.
