# Supplementary material for: Uncovering the balance of forces driving microtubule aster migration in C. elegans zygotes
Source: Nat Commun. 2018 Mar 5;9:938. doi: 10.1038/s41467-018-03118-x (PMC5838244; doi:10.1038/s41467-018-03118-x)
Supplement: Supplementary file 3 — Description of Additional Supplementary Files [file 41467_2018_3118_MOESM3_ESM.pdf]

Description of Additional Supplementary Files

File Name: Supplementary Movie 1
Description: One-cell embryos expressing GFP::TAC-1 imaged using 3D time-lapse DIC and fluorescent microscopy (GFP). Centrosomes (blue and red dots – z-projections) are shown superimposed onto single plane DIC images. Pronuclei are highlighted (blue disc: female pronucleus; red disc: male pronucleus; black crosses: centers – z-projections). Time 0 s is defined as pronuclear meeting; an image stack was captured every 6 seconds. Scale bar: 10 µm.

File Name: Supplementary Movie 2
Description: Pronuclear migration and centration in zyg-12(ct350) goa1/gpa-16(RNAi) embryos. One-cell zyg-12(ct350) goa-1/gpa-16(RNAi) embryos expressing GFP::TAC-1 imaged using 3D time-lapse DIC and fluorescent microscopy (GFP). Centrosomes (blue and red dots – z-projections) are shown superimposed onto single plane DIC images. Pronuclei are highlighted (blue disc: female pronucleus; red disc: male pronucleus; black crosses: centers – z-projections). Time 0 s is defined as half-centration time; an image stack was captured every 12 seconds. Scale bar: 10 µm.

File Name: Supplementary Movie 3
Description: Pronuclear migration and centration in goa-1/gpa16(RNAi) embryos. One-cell goa-1/gpa-16(RNAi) embryos expressing GFP::TAC-1 imaged using 3D timelapse DIC and fluorescent microscopy (GFP). Centrosomes (blue and red dots – zprojections) are shown superimposed onto single plane DIC images. Pronuclei are highlighted (blue disc: female pronucleus; red disc: male pronucleus; black crosses: centers – z-projections). Time 0 s is defined as pronuclear meeting; an image stack was captured every 6. Scale bar: 10 µm.

File Name: Supplementary Movie 4
Description: Pronuclear migration and centration in top-2(it7) goa1/gpa-16(RNAi). One-cell top-2(it7) goa-1/gpa-16(RNAi) embryos expressing GFP::TAC-1 imaged using 3D time-lapse DIC and fluorescent microscopy (GFP). Centrosomes (blue and red dots – z-projections) are shown superimposed onto single plane DIC images. Pronuclei are highlighted (blue disc: female pronucleus; red disc: male pronucleus; black crosses: centers – z-projections). Time 0 s is defined as pronuclear meeting; an image stack was captured every 6. Scale bar: 10 µm

File Name: Supplementary Data 1
Description: Pronuclear and centrosome positions as a function of time for GFP::TAC-1, as well as embryos expressing this fusion protein and treated as follows: goa-1/gpa-16(RNAi), zyg12(ct350) goa-1/gpa-16(RNAi), top-2(it7) goa-1/gpa-16(RNAi) and gpr-1/2(RNAi). Positions are expressed in µm units. The origin of the x- and y-axes is the anterior pole of the embryo, whereas the origin of the z-axis is the first image of each stack. The x-axis is directed from the anterior to the posterior pole and is parallel to the images planes. The zaxis is perpendicular to the images planes.
